# Long-Term Rehabilitation Therapy Is Effective for Physical Function in a Patient With Amyloid Light Chain Amyloidosis Complicated by Nephrotic Syndrome: A Case Report and Literature Review

**DOI:** 10.7759/cureus.64830

**Published:** 2024-07-18

**Authors:** Kenichi Fudeyasu, Yuki Nakashima, Daisuke Iwaki, Koki Fukuhara, Akiko Nagao, Ren Chishaki, Yukio Mikami

**Affiliations:** 1 Division of Rehabilitation, Department of Clinical Practice and Support, Hiroshima University Hospital, Hiroshima, JPN; 2 Department of Dietary Management, Hiroshima University Hospital, Hiroshima, JPN; 3 Department of Hematology and Oncology, Research Institute for Radiation Biology and Medicine (RIRBM), Hiroshima University, Hiroshima, JPN; 4 Department of Rehabilitation Medicine, Hiroshima University Hospital, Hiroshima, JPN

**Keywords:** amyloid light chain (al) amyloidosis, muscle strength, physical therapy, rehabilitation, nephrotic syndrome, al amyloidosis

## Abstract

We report on the rehabilitation of a patient with amyloid light chain (AL) amyloidosis complicated by nephrotic syndrome. Various symptoms produced by AL amyloidosis, including nephrotic syndrome, complicate rehabilitation therapy. In this case report, long-term physical therapy was initiated prior to autologous peripheral blood stem cell transplantation owing to the risk of further decline in physical function due to decreased mobility and physical activity. Patients were instructed on how to perform home exercise therapy. Furthermore, compliance was monitored using a checklist and regular face-to-face feedback. There was no increase in skeletal muscle mass, but improvements in grip strength, lower extremity muscle strength, and phase angle were observed after 24 weeks of physical therapy. Despite the absence of partial remission (urinary protein level of 3.5 g/gCre or higher), nephrotic syndrome demonstrated a trend toward improvement. Since the effectiveness of physical therapy in such patients has not yet been fully established, this report suggests that long-term rehabilitation therapy for physical function in patients with nephrotic syndrome complicated by persistent proteinuria may be effective.

## Introduction

Amyloid light-chain (AL) amyloidosis is a rare disease, with an estimated 3,170 cases reported from 2012 to 2014 [1]. Amyloidosis is an intractable disease in which amyloid proteins with fiber structures are deposited extracellularly in various organs, causing organ damage. Systemic AL amyloidosis had a poor prognosis, with a median survival of only 13 months for patients diagnosed in the early 1990s [2]. However, effective treatments, such as autologous stem cell transplantation and novel therapeutic agents, have recently been introduced, which have improved the survival rate by three to four years [3].

Recently, sarcopenia, which is associated with decreased muscle mass and physical function, has been demonstrated as a prognostic factor in patients indicated for hematopoietic stem cell transplantation (HSCT) (such as AL amyloidosis) [4-6]. In patients undergoing HSCT, sarcopenia is associated with a 2.5-fold increased risk of all-cause mortality one year after HSCT [4]. In patients undergoing allogeneic stem cell transplantation, those with sarcopenia, have a significant three-fold increase in all-cause and non-recurrent mortality [6]. Therefore, we can speculate that improving muscle mass and physical function in patients with AL amyloidosis is crucial.

Rehabilitation therapy, including exercise and nutritional therapy, is important for improving muscle mass and physical function. However, patients with AL amyloidosis exhibit varying symptoms owing to amyloid protein deposition, making rehabilitation therapy challenging. Protein in multiple organs, including the kidneys, heart, liver, gastrointestinal tract, peripheral nerves, and soft tissues [7], causes various symptoms, including heart failure, arrhythmia, dehydration due to diarrhea, low nutrition, orthostatic hypotension due to autonomic dysfunction, and paralysis due to peripheral neuropathy. In particular, the glomeruli in the kidneys are damaged, resulting in nephrotic syndrome with proteinuria as the main symptom [8]. A systematic review of exercise therapy for patients with chronic kidney disease found that exercise therapy does not exacerbate proteinuria [9]. On the other hand, “limit exercise” and “avoid excessive bed rest” to prevent deep vein thrombosis and pulmonary thromboembolism are the only recommendations in nephrotic syndrome, since increased coagulability and blood stasis due to prolonged bed rest are considered risk factors for deep vein thrombosis and pulmonary thromboembolism [10,11]. Thus, the long-term effectiveness of physical therapy for patients with nephrotic syndrome has not been elucidated. Furthermore, no studies have demonstrated the efficacy of exercise therapy for nephrotic syndrome complicated by AL amyloidosis. Herein, we report a case of nephrotic syndrome complicated by AL amyloidosis, in which long-term physical therapy resulted in the improvement of physical function despite persistent proteinuria.

## Case presentation

A 50-year-old man weighing 57.6 kg was diagnosed with AL amyloidosis one year and three months prior to the start of physical therapy. The patient was diagnosed with nephrotic syndrome at another hospital, and two months after the diagnosis, low immunoglobulin levels and autologous peripheral blood stem cells were observed. Autologous peripheral blood stem cells (7.2 x 10^6^/kg) were collected for stem cell transplantation three months prior to the start of physical therapy. The following month, however, the patient required a short-term hospitalization due to dehydration with frequent diarrhea, and stem cell transplantation was postponed.

One month after discharge (week 0), the patient began outpatient physical therapy due to physical dysfunction and persistent limiting impairment in the ability to perform activities of daily living. Daily activities were primarily confined to the home environment. Nutritional guidance was provided by a registered dietician the following month (week four), and ongoing outpatient physical therapy was terminated after six months (week 24) as the patient had developed exercise habits and demonstrated improved physical function.

Diagnostic assessment

To verify the effect of rehabilitation therapy, body composition (including body weight, skeletal muscle mass index (SMI), the extracellular water-to-total body water ratio (ECW/TBW), and phase angle (PhA)) were evaluated via bioelectrical impedance analysis (BIA) measurement using an InBody S10 body composition analyzer (Biospace, Seoul, Korea). Each BIA measurement was performed with the patient in the prone position and included values for appendicular skeletal muscle mass (ASM). Total SMI was calculated based on the diagnostic criteria for sarcopenia according to the Asian Working Group for Sarcopenia (AWGS) 2019 using the following formula: SMI (kg/m^2^) = ASM/height^2^ (m^2^) [12]. The ECW/TBW was calculated as the ratio of the ECW and TBW measurements. The PhA was calculated with resistance (R) and reactance (Xc; measured at 50 kHz) using the following equation: PhA (°) = arctangent(Xc/R) × (180/π).

Hand grip strength (HGS) and knee extension force (KEF) were recorded as physical functions. The HGS was measured in the normal sitting position with elbows flexed to 90° using a Jamar-type digital hand grip dynamometer (MG-4800; CHARDER, Taichung, Taiwan). The KEF was measured using a belt-mounted hand-held dynamometer (μTas F-1; Anima, Tokyo, Japan). The patient was instructed to sit in an end-occupied position with the trunk upright, one lower limb secured to the belt, and the knee joint flexed at 90°. Measurements for both HGS and KEF were performed twice on each side and averaged from the maximum values on each side.

Therapeutic intervention

For physical function improvement, the patient was instructed to perform strength training and increase physical activity and was encouraged to continue this at home. At the beginning of physical therapy (week 0), heel-raising, squatting, and hip abduction exercises were performed for three sessions of 20 times as strength training exercises that can be performed at home to activate the antigravity muscles, mainly in the lower limbs and trunk. He was then instructed to practice these three types of strength training at home for three sessions, 20 times a day. Subsequent four-weekly outpatient physical therapy focused on reviewing strength training methods and providing feedback. As an alternative to aerobic exercise, the patient was encouraged to monitor his physical activity levels and was instructed to gradually increase his physical activity level. Specifically, the patient was instructed to increase his physical activity level by 500 steps every two weeks thereafter, with the first two weeks serving as a baseline. Exercise intensity was assessed using the modified Borg CR10 scale, an 11-point scale ranging from 0 (no exertion) to 10 (maximum exertion); the patient was instructed to aim for a CR10 scale of three to five (moderate to somewhat hard) for their exercise therapy. In addition, body composition and physical function were evaluated eight-weekly, and the results were provided as feedback to motivate the patient to continue the exercise therapy.

Nutritional guidance was provided by a registered dietitian four weeks after the start of physical therapy. Dietary goals were set at an energy intake of 1800 kcal/day, protein intake of 50 g/day, and salt intake of 6 g/day. The patient complained of abnormal taste and loss of appetite and had low zinc levels (47 μg/dL); thus, oral zinc preparation was started.

Follow-up and outcomes

Changes in the body composition, physical function, and various blood parameters over time are shown in Table 1.

**Table 1:** Changes in the body composition, physical function, and various blood parameters over time A 50-year-old man with amyloid light chain amyloidosis complicated by nephrotic syndrome with highly persistent proteinuria received exercise and nutritional therapy as an outpatient. Physical function was assessed every eight weeks during a 24-week follow-up period. The results showed that knee extension force improved from 28.3 kgf to 31.2 kgf, hand grip strength improved from 25.8 kg to 30.0 kg, and phase angle improved from 3.3° to 3.8°. HGS: hand grip strength; KEF: knee extension force; SMI: skeletal muscle mass index; PhA: phase angle; ECW/TBW: extracellular water-to-total body water ratio; Cre: creatinine; BUN: blood urea nitrogen; eGFR: estimated glomerular filtration rate; ALB: albumin; CRP: C-reactive protein

	Week 0	Week 8	Week 16	Week 24
Weight (kg)	57.6	53.7	54.5	58
HGS (kg)	25.75		27.25	30
KEF (kgf)	28.3		28.7	31.2
SMI (kg/m^2^)	7.6	7.3	7.2	7.3
PhA (°)	3.3	3.1	3.5	3.8
ECW/TBW	0.408	0.42	0.413	0.411
Cre (mg/dL)	1.03	0.94	0.98	1.06
BUN (mg/dL)	16.8	16.8	20.6	27.3
eGFR (mL/min/1.73 m^2^)	60	65	64	59
ALB (g/dL)	1.3	1.4	1.7	1.8
Urinary protein (g/gCre)	4.72	3.98	4.19	3.81
CRP (mg/dL)	0.32	<0.02	0.29	0.13

The urinary protein levels remained high during the intervention period. Although they tended to decline, they never fell below <0.3 g/gCre indicating complete remission, or <3.5 g/gCre indicating partial remission.

After nutritional guidance, his diet, which was heavily focused on noodles, improved, and his energy intake became adequate. The patient did not show sufficient improvement in physical function, with a decrease in the PhA at eight weeks and a flat KEF at 16 weeks. Finally, the patient showed increased body weight, HGS, KEF, and PhA, and decreased SMI. The KEF values increased from 28.3 kgf to 31.2 kgf, the HGS values from 25.8 kg to 30.0 kg, and the phase angle from 3.3° to 3.8°. No deep vein thrombosis or pulmonary thromboembolism was observed, and renal function did not deteriorate.

As a treatment strategy, HSCT, which had been postponed owing to concerns about reduced physical function, was reconsidered. Thirteen months after completion of physical therapy (day 570), the patient underwent high-grade melphalan therapy and autologous peripheral blood stem cell transplantation (3.6 x 10^6^/kg). The following month (day 600), he was discharged in good health. The patient is currently alive.

## Discussion

We examined the benefit of rehabilitation therapy for patients with nephrotic syndrome associated with AL amyloidosis. No studies have reported on the safety and efficacy of long-term exercise therapy in patients with nephrotic syndrome who do not show partial remission. Patients with nephrotic syndrome with urinary protein levels higher than 3.5 g/gCre, which does not achieve even partial remission, had demonstrated difficulty improving physical function [13,14]. In the present case, the urinary protein levels were above this cutoff value; nevertheless, patients demonstrated a trend toward physical function improvement. Furthermore, no adverse events, such as deep vein thrombosis, pulmonary thromboembolism, or worsening renal function, were observed, indicating that improvement in body composition and physical function is possible with appropriate long-term exercise and nutritional therapy. The patient provided regular feedback on physical activity and strength training, which could be continued at home, using a checklist. The patient had four-weekly exercise therapy compliance checks during the outpatient rehabilitation sessions as well as eight-weekly body composition and physical function assessments and received feedback on the assessment. A meta-analysis reported motivation as an effective factor for maintaining exercise therapy [15]. In this particular case, regular feedback on compliance with exercise therapy and physical function assessment was deemed to have improved the patient's motivation to continue with the therapy.

Adherence to long-term nutritional and exercise therapy improved lower extremity muscle strength, grip strength, and PhA over time. The improvement in body composition and muscle strength in this case may be due to the effect of exercise therapy in addition to nutritional therapy. In patients with nephrotic syndrome who do not achieve even partial remission and have urinary protein levels above 3.5 g/gCre, the reported intervention period for physical function was five weeks, indicating that it is difficult to improve physical function [13,14]. The present case also showed no improvement in physical function at eight to 16 weeks, but long-term results showed improvements in body composition and physical function. The effect of rehabilitation treatment on patients with nephrotic syndrome who show a trend towards improvement but no partial remission may require more time. In addition, zinc deficiency is likely to be accompanied by a decrease in serum albumin levels [16,17]. Zinc deficiency is associated with taste disorders, and oral administration of zinc preparations has been shown to improve this symptom [18]. In the present case, food intake increased after the patient started taking oral zinc preparations. The patient's initial grip strength was 25.8 kg, which was lower than that of the diagnostic criteria for sarcopenia (28 kg) [12]. However, with continuous nutrition and exercise therapy, his grip strength improved to 30 kg. The 4.2 kg improvement in grip strength in this case is considered clinically significant because a 1 kg improvement is associated with a 5% decrease in all-cause mortality [19]. Moreover, the outcome of the HSCT was concerning owing to the patient's impaired physical function [20]. However, HSCT was successfully completed owing to physical function improvement.

We observed a decrease in SMI and an increase in PhA. No improvement in muscle mass was observed. The ECW/TBW ratio, which reflects edema, was high from the beginning of the intervention, requiring careful interpretation of the SMI results. The presence of edema due to nephrotic syndrome may have led to an overestimation of muscle mass in this case, which has been attributed to BIA muscle mass measurements [21-24], contributing to the lack of improvement in SMI. A high correlation between PhA and the prognosis of various diseases has been reported in recent studies [25,26]. In patients with chronic kidney disease, PhA has been associated with hospitalization-free survival [27]. In addition, PhA has been shown to be strongly associated with physical function, including handgrip strength [27,28], peak torque of the knee extensors [28], and the 4-m gait test [29]. Based on the present findings, PhA may be similarly associated with physical function in patients with nephrotic syndrome with edema.

It is important to note that this report describes the case of a single patient, and caution must be exercised before considering the safety of exercise in patients with amyloidosis. However, as amyloidosis is a rare disease with a small number of cases, it is important to accumulate additional case reports.

## Conclusions

In summary, this patient presented with a nephrotic syndrome that was not in remission (urinary protein level >3.5 g/gCre) , however, he showed improvement in physical function. This case suggests a new challenge in the management of AL amyloidosis with nephrotic syndrome. Although the efficacy of physical therapy for such patients has not yet been fully established, this report suggests that long-term rehabilitation therapy for physical function in patients with nephrotic syndrome complicated by persistent proteinuria may be effective.
